# The First Two Complete Mitochondrial Genomes of Neoephemeridae (Ephemeroptera): Comparative Analysis and Phylogenetic Implication for Furcatergalia

**DOI:** 10.3390/genes12121875

**Published:** 2021-11-24

**Authors:** Ran Li, Zhenxing Ma, Changfa Zhou

**Affiliations:** 1 The Key Laboratory of Jiangsu Biodiversity and Biotechnology, College of Life Sciences, Nanjing Normal University, Nanjing 210023, China; li471329014@163.com (R.L.); 161202021@njnu.edu.cn (Z.M.); 2 School of Life Sciences, Qufu Normal University, Qufu 273165, China

**Keywords:** Furcatergalia, Neoephemeridae, mayfly, mitogenome, phylogeny

## Abstract

Mayflies of the family Neoephemeridae are widespread in the Holarctic and Oriental regions, and its phylogenetic position is still unstable in the group Furcatergalia (mayflies with fringed gills). In the present study, we determined the complete mitogenomes of two species, namely *Potamanthellus edmundsi* and *Pulchephemera projecta*, of this family. The lengths of two mitogenomes were 15,274 bp and 16,031 bp with an A + T content of 73.38% and 73.07%, respectively. Two neoephemerid mitogenomes had a similar gene size, base composition, and codon usage of protein-coding genes (PCGs), and the sequenced gene arrangements were consistent with the putative ancestral insect mitogenomes as understood today. The most variable gene of Furcatergalia mitogenomes was ND2, while the most conserved gene was COI. Meanwhile, the analysis of selection pressures showed that ND6 and ATP8 exhibited a relaxed purifying selection, and COI was under the strongest purifying selection. Phylogenetic trees reconstructed based on two concatenated nucleotide datasets using both maximum likelihood (ML) and Bayesian inference (BI) estimations yielded robust identical topologies. These results corroborated the monophyly of seven studied families and supported the family Leptophlebiidae as being of the basal lineage of Furcatergalia. Additionally, the sister-group relationship of Caenidae and Neoephemeridae was well supported. Methodologically, our present study provides a general reference for future phylogenetic studies of Ephemeroptera at the mitogenome level.

## 1. Introduction

The mitochondrion is an important organelle in metazoan cells as it is mainly involved in life cycle, apoptosis, and metabolism [1]. In most insects, the mitochondrial genome (mitogenome) contains a small double-stranded circular molecule of 14–20 kb in size and has a relatively stable organization and structure [2]. It generally encodes 37 genes including 13 protein-coding genes (PCGs), 22 transfer RNA genes (tRNAs), and two ribosomal RNA genes (rRNAs). In addition, it is composed of a control region (also called A + T-rich region) which contains the initiation sites for transcription and replication [2,3]. Compared to the nuclear genome, the mitogenome possesses multiple obvious advantages, such as maternal inheritance, absence of introns, conserved gene composition, the relatively rare recombination, and high evolutionary rate [4]. Given the vast diversity of insects, mitogenome sequences are usually considered to be effective molecular markers for species identification and play an increasingly important role in intraspecific and interspecific genetic differences, phylogenetic implications, molecular evolution, and phylogeographic studies across various taxa [5,6,7,8]. With the rapid advance of high-throughput sequencers (whole-genome sequencing (WGS) and next generation sequencing (NGS)) lowering the processing requirements and expense of DNA sequencing, increasing numbers of mitogenomes have been obtained in diverse insect orders [9,10,11]. However, the mitogenomes of Ephemeroptera have been limitedly studied and approximately only 50 reliable sequences (with exact Latin name of the species) were made public in the NCBI database (National Center for Biotechnology Information). More importantly, most of these available mitogenomes were mainly sequenced and focused on several families (such as Heptageniidae and Ephemerellidae), and no sequence of the vast majority of mayfly families was reported up to now [4,12]. This unbalanced distribution of mitogenomes has limited our comprehensive understanding of the evolutionary and phylogenetic relationships within Ephemeroptera at the mitogenome level.

**Figure 1:**
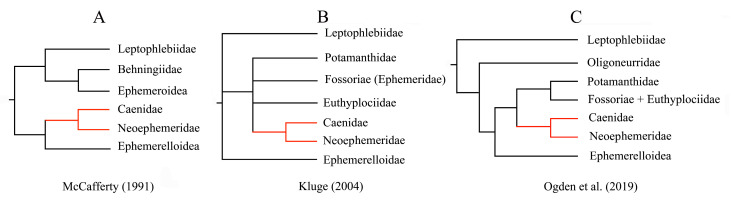
Three hypotheses of the phylogenetic relationships among Furcatergalia. (**A**) McCafferty hypothesis, (**B**) Kluge hypothesis, (**C**) Ogden hypothesis.

Ephemeroptera (mayflies) is one of the most archaic of extant winged insects, originating in the late Carboniferous or early Permian periods (about 300 Mya) [13]. Mayflies occupy freshwater habitats throughout the world, except for Antarctica, with over 3700 described species belonging to 460 genera (42 families) [14,15,16,17]. The well-supported monophyly of Ephemeroptera was estimated in the pterygote insects [18,19]. However, the phylogenetic relationships among mayflies themselves remain partially unresolved [20,21]. Additionally, the higher-level classification and relationships are still unstable, especially for the suborder Furcatergalia, which includes the major clades Leptophlebiidae, Pannota, and the burrowing mayflies [22]. The phylogenetic hypothesis of McCafferty supported the idea that the burrowing mayflies (Behningiidae and Ephemeroidea) were included in a monophyletic group [22]. Leptophlebiidae was hypothesized as a sister group to the group (Behningiidae + Ephemeroidea), with the Pannota (Caenidae, Neoephemeridae, and Ephemerelloidea) sister to this monophyletic group (Figure 1A). The Kluge hypothesis proposed that the burrowing mayflies clustered together with two pannote lineages (Caenidae and Neoephemeridae) [23]. From this system, the infraorder Pannota was not supported as monophyletic (Figure 1B). According to the study based on 440 targeted genomic protein-coding regions (exons), the phylogenetic results were consistent with the Kluge hypothesis that two families (Caenidae and Neoephemeridae) were grouped together with the burrowing mayflies (Figure 1C). Furthermore, this molecular analysis showed that the monophyletic Leptophlebiidae was sister to all other clades within Furcatergalia [21]. However, our previous study using mitogenomes showed that Leptophlebiidae was clustered with the group (Caenidae + Baetidae) [24]. In the newly published research by Xu et al. (2021), the results placed Caenidae as a sister group of (Baetidae + Teloganodidae) and Leptophlebiidae was then consolidated together [25]. Over the past decade, multiple phylogenetic trees using mitogenomes were reconstructed and all of these included only a subset of Ephemeroptera families due to a lack of taxon sampling [24,25]. Until now, the phylogenetic relationships of different families in Furcatergalia remain unresolved at the mitogenome level.

Mayflies of the family Neoephemeridae are widespread in the Holarctic and Oriental regions [26]. So far, 13 species of Neoephemeridae have been described worldwide [27]. Larvae could be found from mountain torrents to large streams and rivers, generally being either clingers on erosional substrates or sprawlers on depositional substrates [26,27]. Studies of Neoephemeridae have mainly focused on the morphological classification and biogeography [26,27,28,29,30]. However, there is no mitogenome sequence of this family available in GenBank. In order to better understand the characteristic of the neoephemerid mitogenome and the phylogenetic relationships within Furcatergalia, we sequenced and analyzed two complete mitogenomes of *Potamanthellus edmundsi* and *Pulchephemera projecta*. Subsequently, we performed comparative analyses of the mitogenome features among Furcatergalia species concerning genomic structure, nucleotide composition, and the secondary structure of tRNAs. In addition, we incorporated the new mitogenome sequences into the Furcatergalia dataset to obtain a more reliable and robust phylogeny to understand the phylogenetic relationships within Furcatergalia.

## 2. Results and Discussion

### 2.1. Features of the Sequenced Mitogenomes

**Figure 2:**
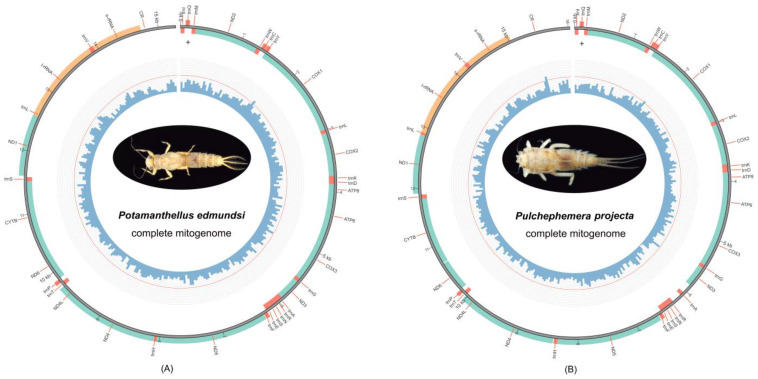
Mitochondrial maps of *P. edmundsi* (**A**) and *P. projecta* (**B**).

A total of 2.82 Gb and 2.25 Gb pair-end clean data from *P. edmundsi* and *P. projecta* were generated by next-generation sequencing on the Illumina platform. The sequencing qualities were high for both mayflies and the Q20 (quality score 20) base percentage of two samples were 98.41 and 98.17, respectively. Both complete mitogenomes were circular double-stranded structures (GenBank accession numbers: OK272542 and OK272543) and the sequences were 15,274 bp (*P. edmundsi*) and 16,031 bp (*P. projecta*) in size (Table 1). Circular maps of two newly sequenced mitogenomes are shown in Figure 2. The finding was comparable to the sequence sizes found for other reported Ephemeroptera complete mitogenomes, which ranged from 14,589 bp of *Alainites yixiani* [31] to 16,616 bp of *Siphluriscus chinensis* [32]. Differences between species were predominantly driven by the overall length of the non-coding regions, especially in the control region (CR; Table 1). The mitogenomes of both neoephemerid species encoded a complete set of 37 genes (13 protein-coding genes (PCGs), two rRNA genes (rrnL and rrnS) and 22 tRNA genes, and a control region (CR; Figure 2 and Table 1). Twenty-three genes (nine PCGs and 14 tRNAs) were located on the majority strand (H-strand), while the other 14 genes (four PCGs, two rRNAs, and eight tRNAs) were oriented on the minority one (L-strand).

Two mitogenomes showed an identical gene order and organization (Figure 2). All genes of both sequences were arranged in the same way without rearrangement or the cracking phenomenon compared with the putative ancestral insect mitogenomes. Of the reported mayfly mitogenomes, several gene rearrangement events have been validated in four families (Siphluriscidae, Baetidae, Heptageniidae, and Ephemerellidae) [31,32,33,34,35]. Previous studies demonstrated that gene rearrangements could effectively resolve the relationships of some groups, which provided additional evidence for phylogenetic reconstruction [35]. For Furcatergalia, the lack of rearrangement was found in limit-sequenced species, except for ephemerellids, up to now [12]. It is apparent that more mitogenomes from diverse groups of Furcatergalia are in demand to well-explore gene rearrangement events in the following studies.

### 2.2. Nucleotide Composition

The alignment analysis showed high sequence similarity across most of the extension of two mitogenomes and the sequence identity was 79.54% (Supplementary Materials, Figure S1). The nucleotide compositions of the two mitogenomes are summarized in Table 2. Both whole mitogenomes were biased in base composition ((A + T)% > (G + C)%), which is consistent with the genomes from other insects [25]. The A + T content of the complete sequence was 73.38% for *P. edmundsi* and 73.07% for *P. projecta* (Table 2). In addition, A + T contents of PCGs (n = 13), rRNAs (n = 2), tRNAs (n = 22), and the control regions (CR) also showed a bias towards A and T nucleotides.

Skew metrics of two mitogenomes showed a negative GC-skew, indicating an obvious bias towards the use of Cs in the complete sequences (Table 2). Meanwhile, the AT-skews were different between two mitogenomes: *P. projecta* was negative and *P. edmundsi* was positive. The comprehensive analysis of all the components showed that most of the AT-skews were negative, except tRNAs. During the progress of replication and transcription, these asymmetries of the nucleotide composition were generally regarded as an indicator for gene direction and replication orientation [36,37].

### 2.3. Protein-Coding Genes

Two newly sequenced mitogenomes comprised the usual set of 13 PCGs: nine PCGs were coded on the H-strand and the other four (ND1, ND4, ND4L, and ND5) were coded on the L-strand (Figure 2 and Table 1). In the mitogenome of *P. projecta*, the total size of all PCGs was 11,223 bp, accounting for 69.38% of the complete sequence. The total size of *P. edmundsi* was 11,246 bp, accounting for 73.62% of the whole mitogenome (Table 2). Both Neoephemeridae species showed a negative AT-skew and positive GC-skew in PCGs, while the third codon position had an A + T content (85.58% and 86.71%) much higher than that of all the positions (73.38% and 72.24%; Table 2). All PCGs of both mitogenomes started with the typical initiation codon ATN (ATG, ATT, and ATC), as seen in other mayflies [4,31,32,33,34]. For the stop codons, most PCGs were terminated by the typical TAN codon (TAG and TAA), apart from several genes (COI, COII, ND5, ND4, CYTB, and ND1) which ended with an incomplete codon T. This incomplete stop codon (T) has been reported in other insect mitogenomes, which connects with transfer RNAs at their 3′ ends, and the processing is presumed to be completed through posttranscriptional polyadenylation [38].

**Figure 3:**
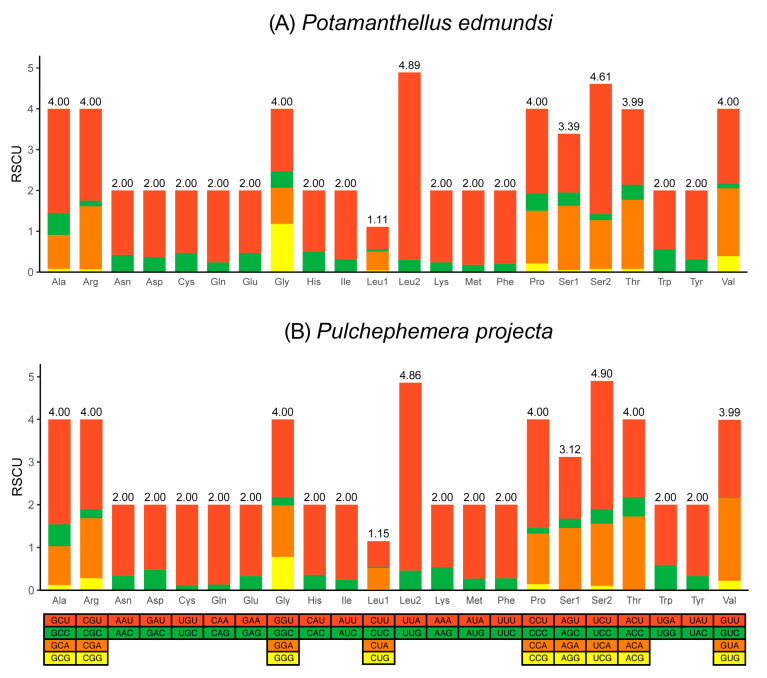
Relative synonymous codon usage (RSCU) in the mitogenomes of *P. edmundsi* (**A**) and *P. projecta* (**B**).

The total numbers of amino acids (without the termination codons) were 3739 (*P. edmundsi*) and 3732 (*P. projecta*), and the codon AGG was not found in *P. edmundsi*. Relative synonymous codon usage (RSCU) values for the 13 PCGs of two mitogenomes are summarized in Figure 3, Tables S1 and S2. Four most frequently used codons UUA (Leucine), UUU (Phenylalanine), AUU (Isoleucine), and AUA (Methionine) were observed in two mitogenomes also fit with some other insects, such as in Coleoptera [9] and Hemiptera [39]. Meanwhile, the RSCU analysis showed that codons were biased to use more A/T at the third codon (Figure 3). Similarly, the biased usage of A + T nucleotides was reflected in the codon frequencies (Table 2).

### 2.4. Ribosomal and Transfer RNA Genes

The size, structure, and distribution of RNA genes between two Neoephemeridae mayflies showed high similarities. The two rRNAs (rrnL and rrnS) were situated between trnL (CUN) and the control region in the L-strand, separated by trnV (Figure 2 and Table 1). The length of rrnL was 1255 bp for *P. edmundsi* and 1250 bp for *P. projecta*, and the size of rrnS was 780 bp for *P. edmundsi* and 781 bp for *P. projecta*.

Two neoephemerid mitogenomes included 22 typical tRNA genes, with the size ranging from 62 bp (trnC and trnP of *P. projecta*) to 71 bp (trnV of *P. edmundsi*). All tRNA secondary structures of two mayflies were inferred and, among all the tRNA genes, 21 could be folded into the regular clover-leaf secondary structures (Figures S2 and S3). The lack of the dihydrouridine (DHU) arm (forming a loop) in trnS (AGN) was found in two species, which is commonly found in other insects, including all other available mitogenomes of mayflies [6,7,8,9]. In the conservative structures, apart from the normal base pairs A-U and G-C, there was also the non-standard pair G-U and other mismatches (U-U and C-A), including 29 mismatches (one C-A and four U-U) found in the stems of *P. edmundsi* and 27 (three U-U) in *P. projecta*. The pair of G-U could be corrected through the editing process and should not affect the transport function [40].

### 2.5. Non-Coding Regions

Two new mitogenomes had gene overlaps ranging from 1 to 35 bp. (*P. edmundsi*: 8 gene junctions and 66 bp overlaps; *P. projecta*: eight gene junctions and 62 bp overlaps). The existing common pairs of gene overlaps, including ATP8−ATP6 (7 bp) and ND4-ND4L (7 bp), were also found in two mitogenomes (Table 1). Additionally, two species also shared the biggest gene overlap of trnY-COI with the size of 35 bp. Apart from the control region (CR), there were 12 and 10 intergenic spacer (IGS) regions, totaling to 402 and 75 bp non-coding bases in *P. projecta* and *P. edmundsi*, respectively. Two new mitogenomes possessed the same four intergenic spacer patterns between ND5-trnH (1 bp), ND4L-trnT (2 bp), trnP-ND6 (2 bp), and ND1-trnL (1 bp). The two biggest IGS regions (162 and 165 bp) between ND3 and trnR (separated by trnA) were found in *P. projecta*. The subsequent Sanger-sequencing confirmed the accuracy of the Illumina sequencing and assembly. Interestingly, none of the other Ephemeroptera mitogenomes showed these IGSs, hence we supposed that this was an isolated incidence during the long evolutionary process of the *P. projecta* mitogenome.

Of the non-coding regions, the putative control region (CR) is usually thought to be the longest one in the whole sequence, which contains signals for the regulation and initiation of mitochondrial DNA transcription and replication [41,42,43]. Like most mayflies, the CRs of neoephemerid mitogenomes were located in the conserved position between two genes: rrnS and trnI. The full length of the CR in *P. edmundsi* was 547 bp, while that of *P. projecta* was 998 bp. In total, the non-coding regions between two mayflies have a significant difference, which is in accordance with other insect mitogenomes considering that the different size of the sequences is generally due to the size variation of the non-coding regions (intergenic spacer region and control region) [44,45].

### 2.6. Comparative Analysis of Furcatergalia Mitogenomes

**Figure 4:**
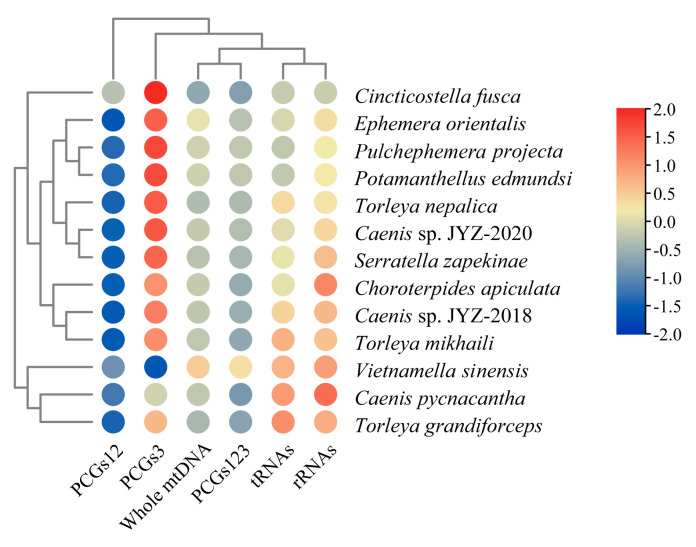
Nucleotide composition of various datasets of 13 Furcatergalia mitogenomes. Hierarchical clustering of mayfly species (y-axis) based on A + T content. PCGs12: all PCGs with only the first and second codon positions, PCGs3: all PCGs with the third codon positions, PCGs123: all PCGs with three codon positions.

**Figure 5:**
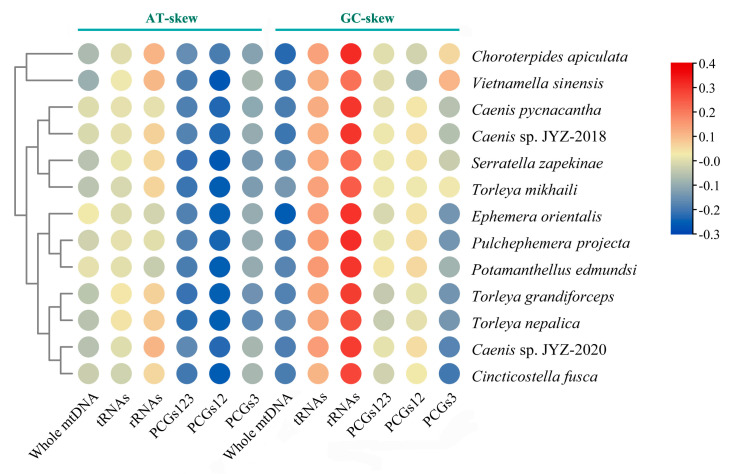
Nucleotide composition of various datasets of 13 Furcatergalia mitogenomes. Hierarchical clustering of mayfly species (y-axis) based on the AT-skew and GC-skew.

**Figure 6:**
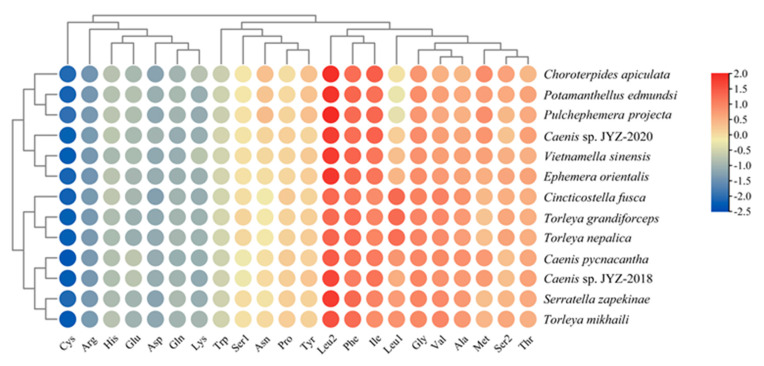
Amino acid composition of all the PCGs of 13 Furcatergalia mitogenomes.

All complete mitogenomes of 13 Furcatergalia mayflies exhibited a AT nucleotide bias (60.32–73.96%) and the AT content (59.60–70.19%) of the PCGs was slightly lower than that of the other regions (Figure 4). AT and GC-skews are a measure of compositional asymmetry. The AT-skew of tRNA and rRNAs were mainly positive and other regions were negative, which indicated that the number of Ts was higher compared to the number of As (Figure 5). For the GC-skew, the whole sequences of 13 species were obviously negative (−0.14 to −0.25), while tRNA and rRNAs were positive. The GC-skews for three matrixes of PCGs were different across species, even among closely related ones, which showed that the GC content was not conservative in Furcatergalia. Beyond that, the AT content of PCGs3 was significantly higher than that of PCGs and PCGs12. The codon usage of all the PCGs of the 13 mayflies was calculated and used to establish the frequently used amino acids. Comparative analysis showed that codon usage patterns and major customarily utilized codons of 13 mitogenomes were highly conservative (Figure 6). The most frequently used amino acids were Leucine2 (Leu2), Phenylalanine (Phe), and Isoleucine (Iso), which were represented by codons with high A or T contents. The AT content and codon usage bias phenomenon could be attributed to the fact that the synonymous codon usage bias in the mitogenomes tends to be from codons ending with A/T to promote transcription [46].

**Figure 7:**
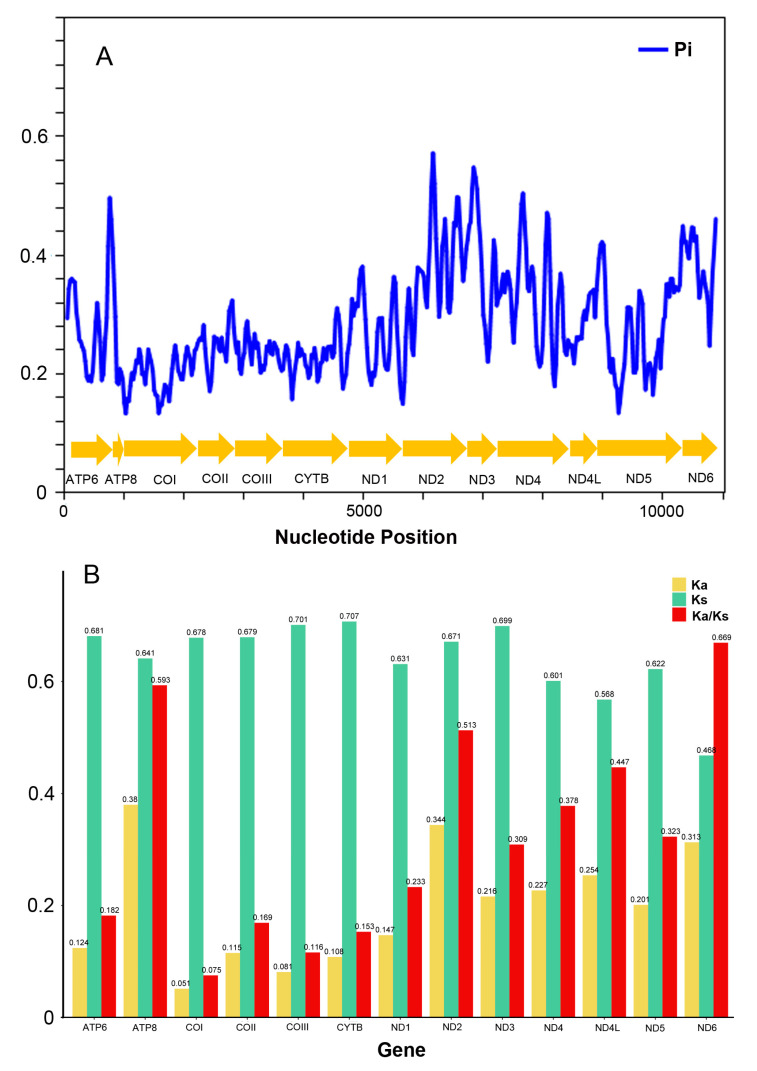
(**A**) Sliding window analysis based on 13 aligned PCGs. The blue line shows the value of the nucleotide diversity Pi. (**B**) Non-synonymous (Ka) to synonymous (Ks) substitution rates of 13 PCGs among 24 Furcatergalia species.

The nucleotide diversity of the PCGs of Furcatergalia mitogenomes is shown in Figure 7A (Sliding window = 100 bp). The most variable region was in ND2, while the most conserved fragment was in COI (lowest Pi), as also found in other mayflies [4,35]. Ka (Nonsynonymous substitutions) and Ks (Synonymous substitutions) are used as indicators of the selectivity constraint [47]. The average Ka/Ks ratios were estimated to investigate evolutionary rates of Furcatergalia PCGs. Ratios ranged from 0.075 of COI to 0.669 of ND6, which suggested that all PCGs were under purifying selection (Ka/Ks < 1). Our results showed that ND6 and ATP8 exhibited relaxed purifying selection, while COI was under the strongest purifying selection (Figure 7B), which showed the gene of Furcatergalia under strong functional restriction.

### 2.7. Phylogenetic Analysis

**Figure 8:**
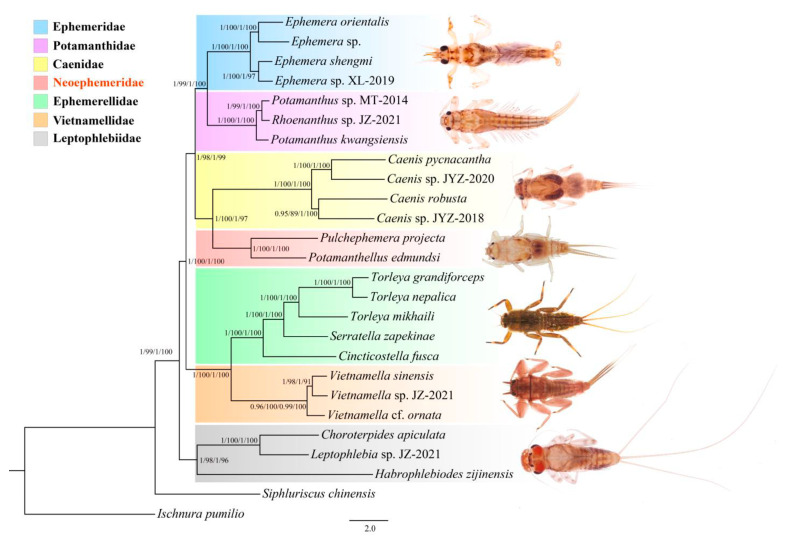
Phylogenetic analyses of Furcatergalia based on the nucleotide datasets of PCG123 and PCG12. Numbers separated by a slash on the nodes represent the posterior probability (PP) and bootstrap value (BV) of PCG123/PCG12.

Phylogenetic trees based on two datasets of 26 specimens (seven families were included as the ingroups) were reconstructed to further investigate the phylogenetic relationships of Furcatergalia. Two datasets used in this study were the matrix PCG123, containing 11,027 sites, and the matrix PCG12, containing 7378 sites. Four phylogenetic trees generated from both analytical methods (BI and ML) had unique topologies, which were combined together (Figure 8). Our phylogenetic analyses were hence considered stable with high nodal support values with mitogenome data.

All analyzed families of Furcatergalia were recovered monophyletic in our phylogenetic trees. Our findings revealed that the family Leptophlebiidae was sister to all other clades, which supported Leptophlebiidae as the basal lineage of Furcatergalia [48]. The trees were then split into two large branches. In the lower branch, the species of Ephemerellidae were clustered together with that of Vietnamellidae. This sister-group relationship of the two families was supported by many previous studies [24,34]. The upper branch was composed of two lineages: the burrowing mayflies (Ephemeridae and Potamanthidae) and the superfamily Caenoidea (containing two families: Neoephemeridae and Caenidae; Figure 8).

The morphological studies revealed that adults of the family Neoephemeridae possessed potamanthid-like wing venation and genitalia but the nymphs possessed caenid-like body and gill patterns [27]. In our analysis, the superfamily Caenoidea (Caenidae + Neoephemeridae) was well supported (Figure 8). The results were in accordance with other molecular research studies based on different markers (molecular and morphology) [14,21,49,50]. Additionally, the topologies of our phylogenetic trees were almost consistent with the Kluge hypothesis and the newly published phylogenetic study for Ephemeroptera with over 440 targeted genomic exons [21]. The phylogenetic relationships of Furcatergalia were well supported in our mitogenomic analyses.

## 3. Materials and Methods

### 3.1. Sample Collection, Identification, and DNA Extraction

Specimens of *P. edmundsi* were obtained from Maolan, Guizhou province of China, in July 2019, while *P. projecta* were obtained from Shangri-La, Yunnan province of China, in April 2021. Fresh samples were originally preserved in 100% ethyl alcohol and cryopreservation at −20 °C was used for further storage of the analyzed samples in the laboratory. All specimens were then morphologically identified by Dr. Changfa Zhou using available taxonomic keys and voucher specimens were deposited in the specimen room of Nanjing Normal University (College of Life Sciences). Total genomic DNA was extracted separately from the whole body of individual sample using the TIANamp Genomic DNA Kit (Tiangen, China) according to the manufacturer’s instructions. The quality of the extract DNA was tested with 1% agarose gels and the concentration was measured using a Nanodrop 2000 spectrophotometer (Thermo, Wilmington, DE, USA).

### 3.2. Whole-Genome Sequencing and Mitogenome Assembly

The DNA samples were remitted to Personal Biotechnology Co., Ltd. (Nanjing, China), for library construction and sequencing. Whole-genome data was generated on the Illumina NovaSeq platform (Illumina, San Diego, CA, USA) with a PE150 strategy (2 × 150 base, paired-end reads). The library (insert size of 400 bp) with two indexes was constructed using the Illumina TruSeq^®^ DNA PCR-Free HT Kit and two libraries were then pooled as well as sequenced together with other projects.

More than 2 GB of raw data of each sample was yielded and filtered into clean reads prior to assembly. The reads with adapter contamination were trimmed with the NGS-Toolkit [51]. Low quality reads (Phred scores < 20) comprised more than 10% of the unknown bases (N) and any duplicated sequences were removed by Prinseq [52]. The de novo assemblies of mitogenomes were performed with clean data using NOVOPlasty 4.2.1 [53]. This software assembles organelle genomes with a seed-and-extend algorithm from whole-genome sequencing data, starting from a related or distant single “seed” sequence and an optional “bait” reference mitogenome. For the mitogenome assembly of two neoephemerid species, we used the COI gene and the complete mitogenome of *Caenis pycnacantha* (Caenidae, GenBank accession number: GQ502451) as the seed and bait reference sequence, respectively. Two assemblies were performed following the developer’s suggestions and using an empirical k-mer size of 33. Subsequently, to investigate the accuracy of next-generation sequencing and de novo assembly, three different fragments (partial COI gene, partial ND5 gene, and fragment containing ND3, trnA, trnR, and trnN genes) were amplified for both species by polymerase chain reaction (PCR) and were then Sanger-sequenced. Six pairs of specific primers for two mitogenomes were designed and are shown in Table S3. The sequencing results were finally aligned with the assembled draft mitogenomes using MEGA X [54].

### 3.3. Mitogenome Annotation and Bioinformatic Analysis

MITOS 2 on the MITOS web server (http://mitos2.bioinf.uni-leipzig.de/ accessed on 11 July 2021) was used for the primary annotation of the two assembled mitogenomes [55]. The invertebrate mitochondrial genetic code was used as the optional setting and BLAST searches in NCBI were used to confirm their accuracy. The start and stop positions of 13 PCGs were manually adjusted and corrected by aligning published data of the Ephemeroptera species in GenBank. Additionally, initiation and termination codons of those PCGs were identified by the ORF Finder (https://www.ncbi.nlm.nih.gov/orffinder/ accessed on 11 July 2021). To verify the results of the primary annotation and to make modifications, ARWEN 1.2 [56] and tRNAScan-SE 1.2.1 [57] were used to identify tRNAs. The secondary structure was also predicted by the MITOS web server [55], followed by manual plotting with Adobe Illustrator CS6. Blastp and Blastn (https://blast.ncbi.nlm.nih.gov/Blast.cgi/ accessed on 11 July 2021) were used to compare the rRNA genes of the mitogenomes of related species reported previously. Intergenic spacers and overlapping regions between genes were estimated manually. The graphical maps of the mitochondrial circular genomes of *P. edmundsi* and *P. projecta* were visualized using the program MitoZ in the visualization module [58]. To assess interspecific variation, pairwise comparison of two new mitogenomes was made with the web tool of mVISTA in the Shuffle-LAGAN mode [59]. Except for two mitogenome sequences of *P. edmundsi* and *P. projecta*, the complete mitogenomes of the other 11 species in Furcatergalia (three species of Caenidae, a single species of Vietnamellidae, five species of Ephemerellidae, a single species of Leptophlebiidae, and a single species of Ephemeridae) were downloaded from GenBank (NCBI). A total of 13 species were used for the analysis of nucleotide composition, codon usage, and relative synonymous codon usage (RSCU) values. Codon usage statistics were calculated using DnaSP 6.0 [60]. The composition skew (base compositional differences) was calculated on the basis of the formula: AT-skew [(A − T)/(A + T)] and GC-skew [(G − C)/(G + C)] [36]. The analysis of the nucleotide diversity with a sliding window of 100 bp and step size of 25 bp was conducted using DnaSP 6.0 [32]. The numbers of the synonymous substitutions (Ks) and non-synonymous substitutions (Ka), and the ratios of Ka/Ks for each PCG were also measured in the DnaSP 6.0 [60].

### 3.4. Phylogenetic Analysis

Including two newly sequenced mitogenomes of *P. edmundsi* and *P. projecta*, a total of 24 mitogenome sequences of Furcatergalia (seven families) were used for phylogenetic analyses (Table S4). Additionally, mitogenomes of two species from the family Siphluriscidae (Ephemeroptera and *S. chinensis*) and Coenagrionidae (Odonata and *Ischnura pumilio*) were used as outgroups. The nucleotide sequences of all 13 PCGs were used for our phylogenetic analyses. Sequences for each PCG were aligned individually with codon-based multiple alignments using the MAFFT 5 algorithm [61] within the TranslatorX online platform (with the L-INS-i strategy and default setting) [62]. The program Gblock 0.91b was used to identify the conserved regions with default settings [63]. The individual aligned sequences were then concatenated by PhyloSuite [64] and split into two datasets: (1) PCG123, including all codon positions of 13 PCGs, and (2) PCG12, including the first and second codon positions of 13 PCGs.

The best partitioning schemes and best-fitting substitution models for two datasets were determined by the program PartitionFinder implemented in the PhyloSuite under a greedy search algorithm with linked branch lengths on the basis of Bayesian information criterion (BIC; Tables S5 and S6) [65]. Two methodologies of Bayesian inference (BI) and maximum likelihood (ML) were selected to reconstruct the phylogenetic trees based on both datasets. For BI analysis, MrBayes 3.2.7a [66] was carried out through the online CIPRES Science gateway [67]. Each run of four, chains was set for 10 million generations with sampling every 1000 generations. The first 25% of the trees of each run were discarded as burn-in during BI analysis and the consensus tree was computed from the remaining trees. ML analysis was performed in RAxML 8.2.12 [68] under a GTRGAMMAI model and branch support for each node was estimated with 1000 bootstrap replicates. The phylogenetic trees were viewed and edited with FigTree 1.4.2 (http://tree.bio.ed.ac.uk/software/figtree/ accessed on 11 July 2021).

## 4. Conclusions

In this study, two neoephemerid mitogenomes were newly sequenced, among which were representatives from the genus *Potamanthellus* (*P. edmundsi*) and *Pulchephemera* (*P. projecta*). Results showed that the sequenced gene arrangements were consistent with the putative ancestral insect mitogenomes as understood today. Two neoephemerid mitogenomes generated in this study had a similar AT nucleotide bias, AT and GC-skews, and a codon usage of PCGs, and were comparable overall to other sequenced Furcatergalia mayflies. The nucleotide diversity of the PCGs of Furcatergalia mitogenomes showed that the most variable gene was ND2, while the most conserved gene was COI. Meanwhile, the analysis of the selection pressures on each gene showed that ND6 and ATP8 exhibited relaxed purifying selection, while COI was under the strongest purifying selection. The comparative analysis could improve our understanding of the evolution of mayfly mitogenomes. Phylogenetic analyses based on PCGs from the mitogenomes of 26 species clarified the phylogenetic relationships of Furcatergalia. The results corroborated the monophyly of the seven families and supported the family Leptophlebiidae as the basal lineage of Furcatergalia. Additionally, the sister-group relationship of Caenidae and Neoephemeridae was well supported at the mitogenome level. Our findings also suggested that the mitogenome sequences were effective molecular markers to study the phylogenetic relationships within Furcatergalia.

## Figures and Tables

**Table 1:** Features of the complete mitogenomes of *P. edmundsi* and *P. projecta*.

Gene	Strand	Position		Intergenic Nucleotides		Codon		Anticodon
		Ppr	Ped	Ppr	Ped	Ppr	Ped	
trnI	J	1–65	1–65	0	0			GAU
trnQ	N	80–148	78–146	14	12			UUG
trnM	J	161–225	180–243	12	33			CAU
ND2	J	226–1236	244–1266	0	0	ATT/TAA	ATT/TAA	
trnW	J	1239–1306	1265–1332	2	−2			UCA
trnC	N	1299–1360	1325–1387	−8	−8			GCA
trnY	N	1361–1424	1392–1454	0	4			GUA
COI	J	1390–2956	1420–2986	−35	−35	ATT/T	ATT/T	
trnL	J	2957–3022	2988–3052	0	1			UAA
COII	J	3023–3710	3054–3741	0	1	ATG/T	ATG/T	
trnK	J	3711–3779	3742–3810	0	0			CUU
trnD	J	3780–3846	3811–3876	0	0			GUC
ATP8	J	3847–4011	3877–4044	0	0	ATT/TAA	ATC/TAA	
ATP6	J	4005–4682	4038–4715	−7	−7	ATG/TAA	ATG/TAA	
COIII	J	4682–5470	4715–5503	−1	−1	ATG/TAA	ATG/TAA	
trnG	J	5469–5531	5503–5566	−2	−1			UCC
ND3	J	5532–5885	5567–5920	0	0	ATT/TAA	ATT/TAG	
trnA	J	6048–6112	5919–5981	162	−2			UGC
trnR	J	6278–6340	5982–6044	165	0			UCG
trnN	J	6341–6405	6045–6109	0	0			GUU
trnS	J	6404–6467	6108–6172	−2	−2			GCU
trnE	J	6468–6532	6173–6236	0	0			UUC
trnF	N	6534–6596	6237–6300	1	0			GAA
ND5	N	6597–8331	6301–8035	0	0	ATG/T	ATG/T	
trnH	N	8333–8395	8037–8099	1	1			GUG
ND4	N	8396–9737	8100–9441	0	0	ATG/T	ATG/T	
ND4L	N	9731–10,027	9435–9731	−7	−7	ATG/TAA	ATG/TAA	
trnT	J	10,030–10,095	9734–9799	2	2			UGU
trnP	N	10,096–10,157	9800–9862	0	0			UGG
ND6	J	10,160–10,675	9865–10383	2	2	ATT/TAA	ATT/TAA	
CYTB	J	10,688–11,822	10383–11517	12	−1	ATG/T	ATG/T	
trnS	J	11,823–11,892	11518–11587	0	0			UGA
ND1	N	11,921–12,866	11606–12556	28	18	ATG/T	ATG/TAA	
trnL	N	12,868–12,932	12558–12621	1	1			UAG
rrnL	N	12,933–14,182	12622–13876	0	0			
trnV	N	14,183–14,252	13877–13947	0	0			UAC
rrnS	N	14,253–15,033	13948–14727	0	0			
CR	J	15,034–16,031	14728–15274	0	0			

**Table 2:** Nucleotide composition of the mitogenomes of *P. edmundsi* and *P. projecta*.

Species	Region	Size (bp)	T (%)	C (%)	A (%)	G (%)	A + T (%)	G + C (%)	AT-Skew	GC-Skew
*P. projecta*	Total genome	16,031	37.51	15.74	35.87	10.88	73.38	26.62	−0.02	−0.18
	PCGs123	11,223	42.80	13.49	29.75	13.95	72.56	27.44	−0.18	0.02
	PCGs12	7464	40.72	16.17	25.23	17.89	65.94	34.06	−0.23	0.05
	PCGs3	3732	46.95	8.23	38.64	6.19	85.58	14.42	−0.10	−0.14
	rRNAs	2031	37.52	8.47	37.81	16.20	75.33	24.67	0.00	0.31
	tRNAs	1439	35.93	11.67	36.62	15.77	72.55	27.45	0.01	0.15
	CR	998	44.16	11.31	38.32	6.20	82.48	17.52	−0.07	−0.29
*P. edmundsi*	Total genome	15,274	36.20	15.83	36.87	11.10	73.07	26.93	0.01	−0.18
	PCGs123	11,246	42.94	13.41	29.30	14.35	72.24	27.76	−0.19	0.03
	PCGs12	7478	40.56	16.57	24.35	18.52	64.91	35.09	−0.25	0.06
	PCGs3	3739	47.69	7.19	39.02	6.10	86.71	13.29	−0.10	−0.08
	rRNAs	2035	38.87	8.60	36.51	16.02	75.38	24.62	−0.03	0.30
	tRNAs	1437	35.98	11.69	36.40	15.94	72.37	27.63	0.01	0.15
	CR	548	42.28	15.33	31.36	11.02	73.65	26.35	−0.15	−0.16

## Data Availability

All data is contained within the article.

## Supplementary Materials

The following are available online at https://www.mdpi.com/article/10.3390/genes12121875/s1, Figure S1: Alignment of the mitogenome sequences of *P. edmundsi* and *P. projecta*; Figure S2: The putative tRNA second structure for the *P. edmundsi* mitogenome; Figure S3: The putative tRNA second structure for the *P. projecta* mitogenome; Table S1: Codon number and RSCU in the mitogenome of *P. edmundsi*; Table S2: Codon number and RSCU in the mitogenome of *P. projecta*; Table S3: The specific primers for the mitogenomes of *P. edmundsi* (PE) and *P. projecta* (PP) used in this study; Table S4: The species information used in this study; Table S5: Partition schemes and best-fitting models selected in the PCG123 dataset; and Table S6: Partition schemes and best-fitting models selected in the PCG12 dataset.
