# Supplementary material for: The First Two Complete Mitochondrial Genomes of Neoephemeridae (Ephemeroptera): Comparative Analysis and Phylogenetic Implication for Furcatergalia
Source: Genes (Basel). 2021 Nov 24;12(12):1875. doi: 10.3390/genes12121875 (PMC8702025; doi:10.3390/genes12121875)
Supplement: Supplementary file 1 [file genes-12-01875-s001.zip › genes-1431750-supplementary.pdf]

Supplementary Materials:

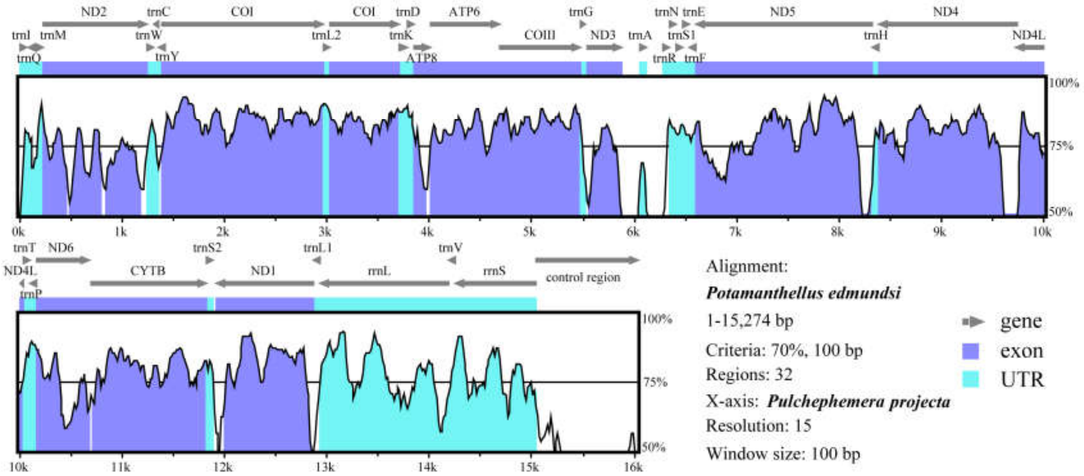

Figure S1. Alignment of the mitogenome sequences of *P*. *edmundsi* and *P*. *projecta*.

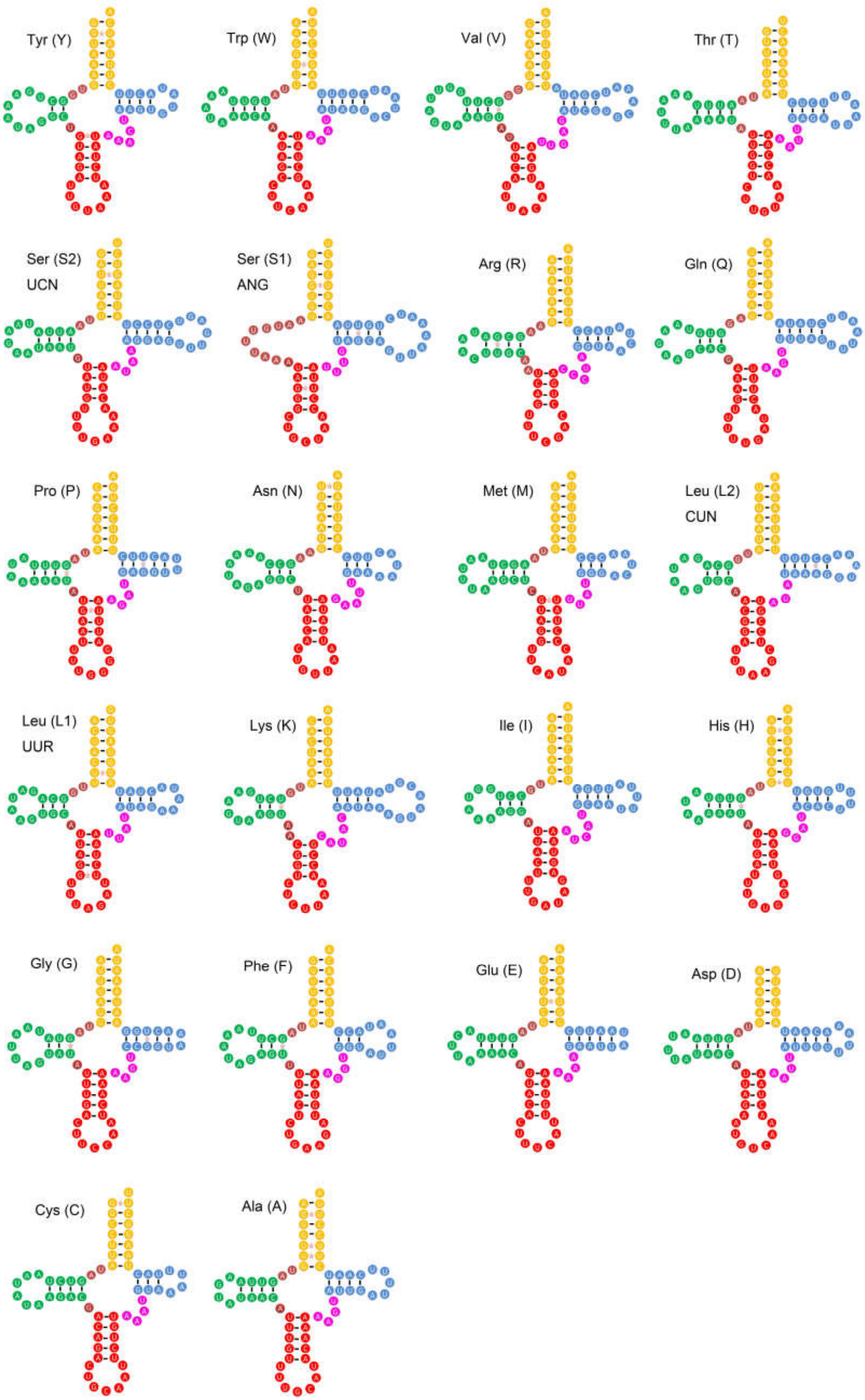

Figure S2. The putative tRNA second structure for *P. edmundsi*

mitogenome.

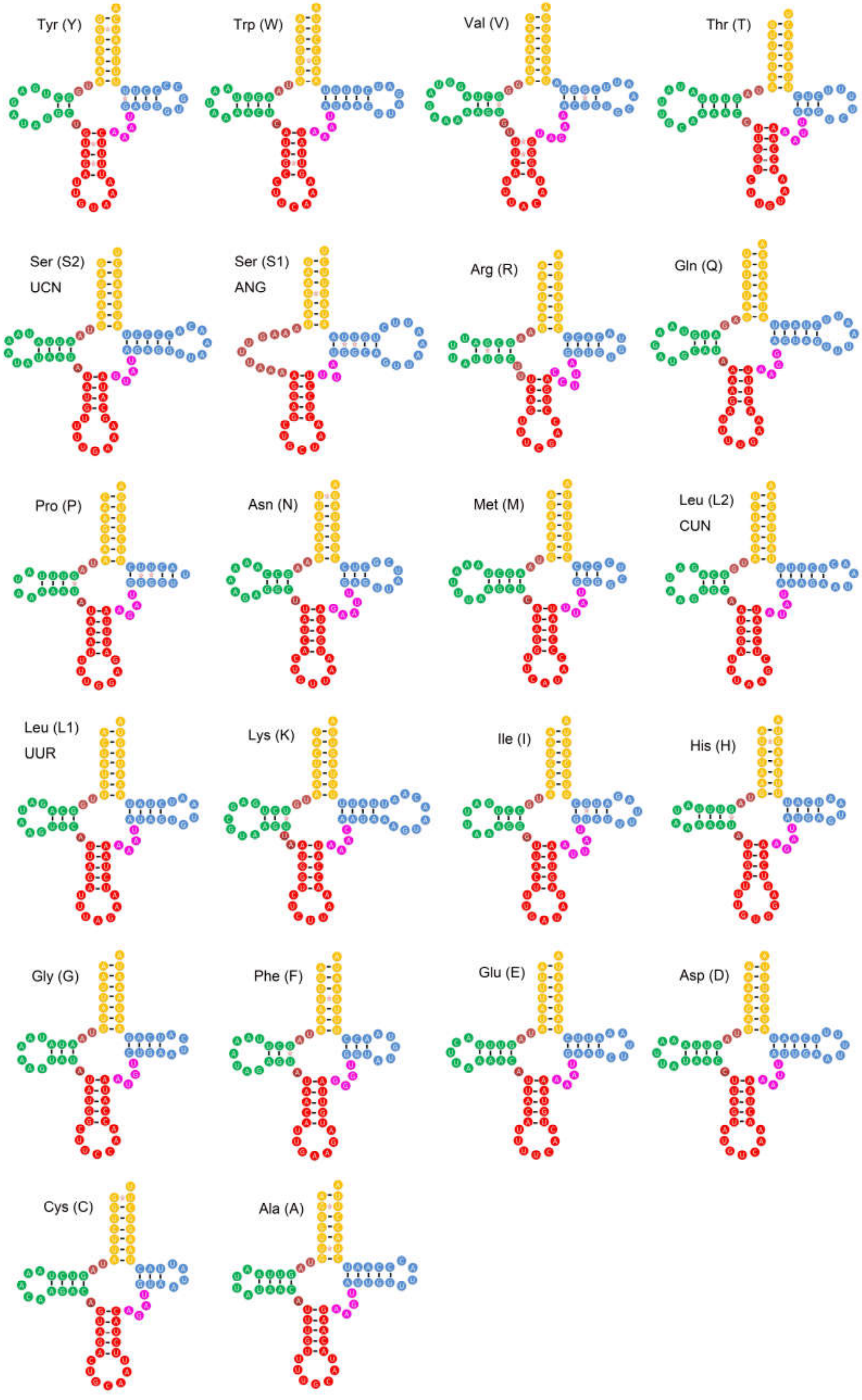
Tyr (Y)
Trp (W)
Val (V)
Thr (T)
Ser (S2)
UCN
Ser (S1)
ANG
Arg (R)
Gln (Q)
Pro (P)
Asn (N)
Met (M)
Leu (L2)
CUN
Leu (L1)
UUR
Lys (K)
Ile (I)
His (H)
Gly (G)
Phe (F)
Glu (E)
Asp (D)
Cys (C)
Ala (A)

Figure S3. The putative tRNA second structure for *P. projecta* mitogenome.

Table S1. Codon number and RSCU in the mitogenome of *P. edmundsi*.

Codon	Count	RSCU	Codon	Count	RSCU	Codon	Count	RSCU	Codon	Count	RSCU
UUU(F)	283	1.72	UCU(S)	125	3.01	UAU(Y)	140	1.67	UGU(C)	33	1.89
UUC(F)	46	0.28	UCC(S)	14	0.34	UAC(Y)	28	0.33	UGC(C)	2	0.11
UUA(L)	458	4.4	UCA(S)	60	1.45	UAA(*)	0	0	UGA(W)	70	1.41
UUG(L)	48	0.46	UCG(S)	4	0.1	UAG(*)	0	0	UGG(W)	29	0.59
CUU(L)	62	0.6	CCU(P)	90	2.54	CAU(H)	69	1.64	CGU(R)	30	2.11
CUC(L)	3	0.03	CCC(P)	5	0.14	CAC(H)	15	0.36	CGC(R)	3	0.21
CUA(L)	51	0.49	CCA(P)	42	1.18	CAA(Q)	73	1.87	CGA(R)	20	1.4
CUG(L)	3	0.03	CCG(P)	5	0.14	CAG(Q)	5	0.13	CGG(R)	4	0.28
AUU(I)	268	1.76	ACU(T)	97	1.83	AAU(N)	138	1.66	AGU(S)	60	1.45
AUC(I)	36	0.24	ACC(T)	24	0.45	AAC(N)	28	0.34	AGC(S)	9	0.22
AUA(M)	189	1.73	ACA(T)	90	1.7	AAA(K)	51	1.46	AGA(S)	60	1.45
AUG(M)	30	0.27	ACG(T)	1	0.02	AAG(K)	19	0.54	AGG(S)	0	0
GUU(V)	98	1.81	GCU(A)	124	2.46	GAU(D)	54	1.52	GGU(G)	112	1.82
GUC(V)	1	0.02	GCC(A)	26	0.51	GAC(D)	17	0.48	GGC(G)	12	0.2
GUA(V)	105	1.94	GCA(A)	46	0.91	GAA(E)	70	1.67	GGA(G)	74	1.2
GUG(V)	12	0.22	GCG(A)	6	0.12	GAG(E)	14	0.33	GGG(G)	48	0.78

Table S2. Codon number and RSCU in the mitogenome of *P. projecta*.

Codon	Count	RSCU	Codon	Count	RSCU	Codon	Count	RSCU	Codon	Count	RSCU
UUU(F)	290	1.8	UCU(S)	126	3.19	UAU(Y)	145	1.69	UGU(C)	29	1.53
UUC(F)	32	0.2	UCC(S)	6	0.15	UAC(Y)	27	0.31	UGC(C)	9	0.47
UUA(L)	473	4.59	UCA(S)	47	1.19	UAA(*)	0	0	UGA(W)	69	1.44
UUG(L)	31	0.3	UCG(S)	3	0.08	UAG(*)	0	0	UGG(W)	27	0.56
CUU(L)	57	0.55	CCU(P)	77	2.07	CAU(H)	65	1.51	CGU(R)	32	2.25
CUC(L)	6	0.06	CCC(P)	16	0.43	CAC(H)	21	0.49	CGC(R)	2	0.14
CUA(L)	47	0.46	CCA(P)	48	1.29	CAA(Q)	65	1.76	CGA(R)	22	1.54
CUG(L)	4	0.04	CCG(P)	8	0.21	CAG(Q)	9	0.24	CGG(R)	1	0.07
AUU(I)	281	1.69	ACU(T)	94	1.85	AAU(N)	140	1.58	AGU(S)	57	1.44
AUC(I)	51	0.31	ACC(T)	19	0.37	AAC(N)	37	0.42	AGC(S)	13	0.33
AUA(M)	226	1.82	ACA(T)	86	1.69	AAA(K)	59	1.76	AGA(S)	62	1.57
AUG(M)	22	0.18	ACG(T)	4	0.08	AAG(K)	8	0.24	AGG(S)	2	0.05
GUU(V)	94	1.83	GCU(A)	123	2.55	GAU(D)	53	1.63	GGU(G)	89	1.53
GUC(V)	6	0.12	GCC(A)	26	0.54	GAC(D)	12	0.37	GGC(G)	24	0.41
GUA(V)	85	1.66	GCA(A)	40	0.83	GAA(E)	62	1.53	GGA(G)	51	0.88
GUG(V)	20	0.39	GCG(A)	4	0.08	GAG(E)	19	0.47	GGG(G)	69	1.18

Table S3. Specific primers for the mitogenomes of *P. edmundsi* (PE) and *P. projecta* (PP) used in this study.

No.	Primers (5'-3')
PE-COI-J	GCCGGTACAGGATGAACTG
PE-COI-N	CTAAAGCCCATAGTAAAGA
PE-ND5-J	GACAGCCTTATCTAACCGT
PE-ND5-N	ATACCCCAATCAACCACCT
PE-R-J	CCTGACCCTAGGTAACTATACCCT
PE-R-N	GTATAGATATGGGTTGGAGAGA
PP-COI-J	ATACCCTCCCTTAGCCGCA
PP-COI-N	CAACACCCGTTAATCCCCC
PP-ND5-J	TCTAACCGTATTGGAGATGTG
PP-ND5-N	CAAGTATTAATTTGGAGCCCT
PP-R-J	GTCTGAATCATCAGAATAAGTA
PP-R-N	TACTTCTAACCGGAGCGATAACTC

Table S4. The species information used in this study.

Family	Species	GenBank #
Caenidae	*Caenis pycnacantha*	GQ502451
	Caenis robusta	MT628575
	Caenis sp. JYZ-2018	MG910499
	Caenis sp. JYZ-2020	MN356096
Ephemeridae	*Ephemera orientalis*	EU591678
	Ephemera shengmi	MF352161

	Ephemera sp.	MF352156
	Ephemera sp. XL-2019	MK951659
Leptophlebiidae	*Habrophlebiodes zijinensis*	GU936203
	Leptophlebia sp. JZ-2021	MF352160
	Choroterpides apiculata	MN807287
Neoephemeridae	*Pulchephemera projecta*	this study
	Potamanthellus edmundsi	this study
Potamanthidae	*Potamanthus kwangsiensis*	MF352158
	Potamanthus sp. MT-2014	KM244674
	Rhoenanthus sp. JZ-2021	MF352145
Ephemerellidae	*Serratella zapekinae*	MT274130
	Cincticostella fusca	MT535767
	Torleya grandiforceps	MT274131
	Torleya mikhaili	MT535766
	Torleya nepalica	MT274132
Vietnamellidae	*Vietnamella dabieshanensis*	HM067837
	Vietnamella sp. JZ-2021	MF352146
	Vietnamella sp. MT-2014	KM244655
Siphluriscidae	*Siphluriscus chinensis*	HQ875717
Coenagrionidae	*Ischnura pumilio*	KC878732

Table S5. Partition schemes and best-fitting models selected in PCG123 dataset.

	**Nucleotide sequence alignment**	
**Subset**	**Partition name**	**Best model**
Partition 1	COII_pos1, COIII_pos1, Cytb_pos1, ATP6_pos1	GTR+I+G
Partition 2	COI_pos2, Cytb_pos2, COIII_pos2, COII_pos2, ATP6_pos2	TVM+I+G
Partition 3	ND6_pos3, ND3_pos3, ATP8_pos3,	GTR+G
Partition 4	ATP8_pos1, ND2_pos1, ND6_pos1	GTR+I+G
Partition 5	ATP8_pos2, COI_pos1, ND6_pos2, ND2_pos2	GTR+I+G
Partition 6	COI_pos3	HKY+I+G
Partition 7	Cytb_pos3, COII_pos3, COIII_pos3	GTR+I+G
Partition 8	ND1_pos1, ND5_pos1, ND4_pos1, ND3_pos1, ND4_pos2	GTR+I+G
Partition 9	ND4L_pos2, ND3_pos2, ND1_pos2, ND5_pos2, ND4L_pos1	GTR+I+G
Partition 10	ND5_pos3, ND1_pos3, ND4L_pos3, ND4_pos3	GTR+G
Partition 11	ND2_pos3, ATP6_pos3	HKY+I+G

Table S6. Partition schemes and best-fitting models selected in PCG12 dataset.

	**Nucleotide sequence alignment**	
**Subset**	**Partition name**	**Best model**
Partition 1	Cytb_pos1, ATP6_pos1, COII_pos1, COIII_pos1, COII_pos2, ATP6_pos2	GTR+I+G
Partition 2	COI_pos2, COIII_pos2, Cytb_pos2, ND2_pos1	TVM+I+G
Partition 3	ND3_pos1, ATP8_pos1, ND6_pos1, ND6_pos2	GTR+I+G
Partition 4	ATP8_pos2, ND2_pos2	GTR+G
Partition 5	COI_pos1, ND4_pos2, ND5_pos2	GTR+I+G
Partition 6	ND1_pos1, ND5_pos1, ND4L_pos1, ND4_pos1	GTR+I+G
Partition 7	ND4L_pos2, ND3_pos2, ND1_pos2,	TRN+I+G
